# Case Report: Psychological acupuncture for severe cynophobia with comorbid PTSD

**DOI:** 10.3389/fpsyt.2026.1753017

**Published:** 2026-03-03

**Authors:** Zixuan Wang, Wenchao Dan, Siqing Wang, Shouchun Zhang, Guangzhong Zhang

**Affiliations:** 1 Dermatological Department, Beijing Hospital of Traditional Chinese Medicine, Capital Medical University, Beijing, China; 2 Department of Acupuncture, Traditional Chinese Medicine, First Affiliated Hospital of Jiaxing University, Jiaxing, Zhejiang, China; 3 Beijing Zhongren International Institute of Psychological Medicine, Beijing, China

**Keywords:** case report, integrative medicine, mind-body therapy, post-traumatic stress disorder, psychological acupuncture, specific phobia

## Abstract

**Objective:**

Post-traumatic stress disorder (PTSD) with comorbid specific phobia represents a challenging clinical condition, particularly when patients decline pharmacological or trauma-focused psychotherapeutic interventions due to fear of re-traumatization or intolerance to adverse effects. This report describes a unique case of severe cynophobia with comorbid PTSD successfully treated using psychological acupuncture, an integrative intervention combining guided trauma recall with fingertip acupoint tapping.

**Methods:**

A 63-year-old woman developed intense fear of dogs, intrusive nightmares, avoidance of outdoor activities, hypervigilance, and severe insomnia following a dog attack. The patient met DSM-5 diagnostic criteria for PTSD and specific phobia (cynophobia) and refused standard psychiatric treatments. Psychological acupuncture was administered in two 40-minute sessions over a two-week interval, incorporating relaxation training, guided trauma recall, and rhythmic fingertip tapping (4–5 Hz) on selected acupoints associated with emotional regulation. Clinical outcomes were assessed using the Subjective Units of Distress scale (SUDs), Insomnia Severity Index (ISI), and PTSD Checklist for DSM-5 (PCL-5).

**Results:**

Marked clinical improvement was observed following the intervention. The patient’s SUDs score decreased from 10 to 3, ISI score from 23 to 7, and PCL-5 score from 54 to 20. Improvements in fear reactivity, sleep quality, and avoidance behavior occurred rapidly after treatment and were maintained at the 3-month follow-up. No adverse events were reported.

**Conclusion:**

This case suggests that psychological acupuncture may serve as a rapid, safe, and well-tolerated integrative therapeutic option for patients with trauma-related specific phobia and comorbid PTSD who decline conventional treatments. The findings highlight the potential role of combined cognitive-emotional activation and somatic modulation in alleviating trauma-related symptoms and support further investigation of this approach in controlled clinical studies.

## 1 Introduction

Specific phobia and post-traumatic stress disorder (PTSD) often co-occur, creating mutual symptom exacerbation in affected individuals. Epidemiological data suggest that the lifetime prevalence of specific phobia is approximately 7-9% in the general population, whereas PTSD affects about 3-4% of adults worldwide (1). Cynophobia is a well-recognized subtype of specific phobia characterized by an excessive and persistent fear of dogs. When cynophobia coexists with PTSD, patients may experience intrusive recollections, nightmares, hypervigilance, avoidance of outdoor environments, and marked sleep disturbance, resulting in substantial functional impairment (2).

Standard treatments for phobia and PTSD include pharmacotherapy (e.g., selective serotonin reuptake inhibitors, benzodiazepines, prazosin) and evidence-based psychotherapies such as cognitive behavioral therapy (CBT), eye movement desensitization and reprocessing (EMDR), and exposure-based interventions (3, 4). However, clinical uptake is often limited by adverse effects, high dropout rates, and patient resistance, particularly among individuals who fear symptom exacerbation or re-traumatization during trauma-focused therapy (5, 6). These barriers underscore the need for acceptable integrative options for patients who refuse or are unable to tolerate conventional treatments (7, 8).

Acupuncture and related somatosensory interventions have emerged as promising complementary strategies for psychiatric conditions (9, 10). Reviews and clinical studies suggest potential benefits for anxiety, insomnia, and PTSD, possibly via modulation of hypothalamic-pituitary-adrenal axis activity and autonomic balance (11–13). Within traditional Chinese medicine (TCM), fear- and anxiety-related symptoms are commonly conceptualized as disturbances of the Shen (spirit/mind) and dysregulation of Qi and blood, with treatment principles emphasizing calming the Shen and restoring internal harmony (14). Nevertheless, reports addressing acupuncture-based approaches for trauma-related phobias remain scarce.

Psychological acupuncture is an emerging, practice-based intervention in China that combines brief, therapist-guided trauma recall with rhythmic fingertip tapping/pressure on selected acupoints. Conceptually, it aligns with bifocal trauma-processing approaches, which pair controlled memory activation with concurrent somatosensory stimulation, as described in the acupoint-tapping literature (e.g., EFT) (9). Neuroimaging findings in phobic populations suggest that tapping during fear-related tasks may be associated with changes in limbic-memory networks, providing a plausible but indirect mechanistic context (15). The technique has been incorporated into the National Health Commission’s Technical Specifications for Traditional Chinese Medicine Psychological Therapies (2023) (16).

To our knowledge, no published case has described psychological acupuncture for severe cynophobia with comorbid PTSD. This report presents such a case, illustrating clinical application and feasibility in a patient who refused pharmacological and trauma-focused psychotherapies, and aims to generate hypotheses for future controlled studies rather than to establish therapeutic efficacy.

## 2 Case description

A 63-year-old retired woman was referred to our outpatient clinic in July 2025 for persistent fear of dogs accompanied by post-traumatic stress symptoms. One year earlier, she had been attacked by a neighborhood dog and subsequently developed intense fear when encountering dogs, even at a distance. Her symptoms included recurrent nightmares, intrusive flashbacks, hypervigilance, irritability, severe insomnia, and marked avoidance of outdoor environments, resulting in reduced physical activity and social engagement. Prior to the incident, she reported normal sleep (6–7 h/night), regular outdoor activity, and no history of anxiety, phobia, or trauma-related symptoms.

Her medical history was notable only for well-controlled mild hypertension. There was no family history of psychiatric disorders, and she denied alcohol or substance use. Mental status examination revealed heightened anxiety, exaggerated startle response, and prominent avoidance behaviors. She met DSM-5 criteria for both specific phobia (cynophobia) and PTSD. PTSD was diagnosed based on persistent intrusion symptoms (nightmares and flashbacks), avoidance, hyperarousal, and sleep disturbance lasting more than 12 months after the traumatic event. Specific phobia was diagnosed due to disproportionate fear of dogs with immediate anxiety responses and avoidance. All clinical scales (SUDs, ISI, PCL-5) were administered and rated by the treating clinician, with SUDs based on patient self-report.

The patient had previously sought psychiatric care but declined pharmacological treatment, including selective serotonin reuptake inhibitors and benzodiazepines, due to concerns about adverse effects and dependence. She also refused trauma-focused cognitive behavioral therapy because of the fear of distress exacerbation. At presentation, her SUDs score was 10, PCL-5 score was 54, and ISI score was 23, with reported sleep duration of only 2–3 h per night.

From a traditional Chinese medicine perspective, her condition was categorized as jing kong (fright syndrome) (17), characterized by disturbance of the Heart Shen, depletion of Kidney essence, and Liver Qi stagnation. The therapeutic principle emphasized calming the Shen and harmonizing Qi.

The patient consented to psychological acupuncture, an emerging integrative intervention delivered by a single clinician trained in both acupuncture and trauma-informed psychological techniques. Each session began with brief relaxation training, followed by guided recall of the core traumatic image while distress was rated on a 0–10 scale.

**Figure 1:**
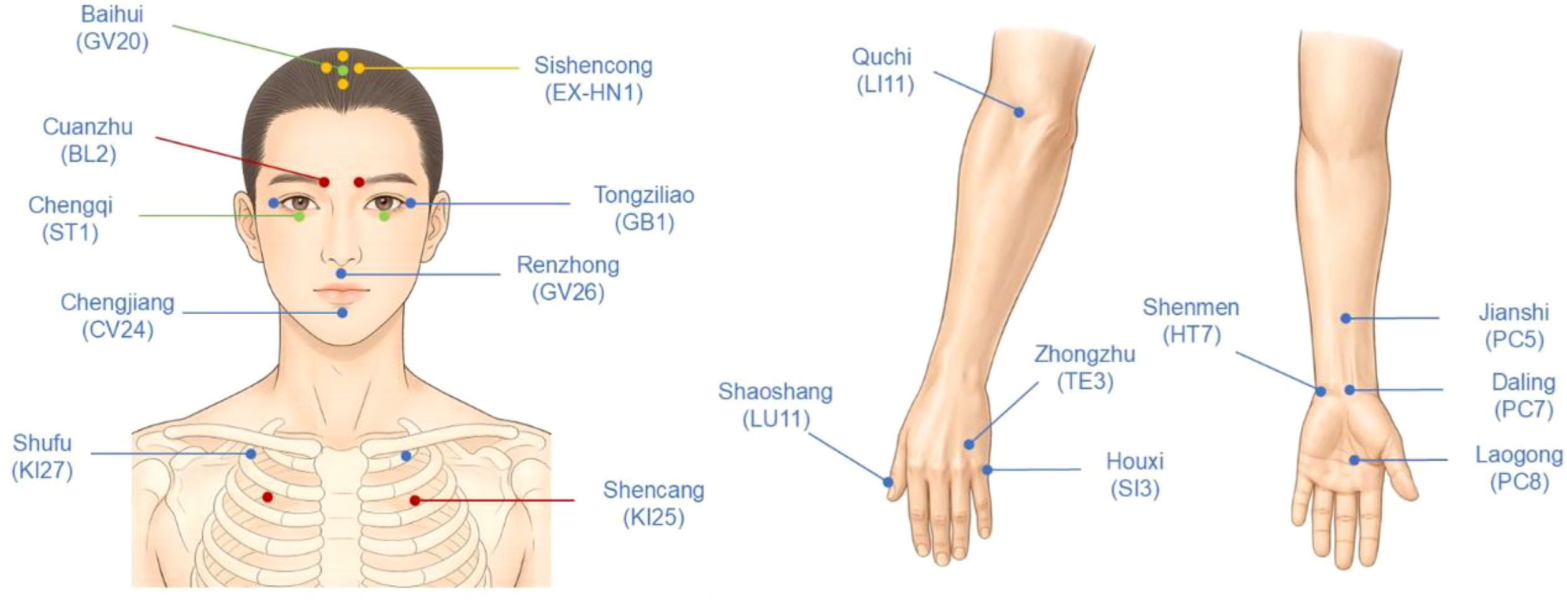
Anatomical locations of acupoints used in the psychological acupuncture intervention.

Rhythmic fingertip tapping (4–5 Hz) was applied in a predefined sequence of acupoints, including Baihui (GV20), Sishencong (EX-HN1), Cuanzhu (BL2), Tongziliao (GB1), Chengqi (ST1), Renzhong (GV26), Chengjiang (CV24), Shufu (KI27), Shencang (KI25), Quchi (LI11), Houxi (SI3), Jianshi (PC5), Daling (PC7), Shenmen (HT7), Laogong (PC8), Shaoshang (LU11), and Zhongzhu (TE3). Acupoint nomenclature and locations followed WHO standards (18), and anatomical locations are illustrated in **Figure 1**.

The tapping sequence was organized by functional grouping rather than anatomical order. Stimulation began with central regulatory points (e.g., GV20, EX-HN1) to promote calming and attentional stabilization, followed by points related to emotional and autonomic regulation (e.g., HT7, PC5). Peripheral points were subsequently engaged to support grounding and reduce somatic hyperarousal during trauma recall, allowing controlled activation of traumatic memory.

At the end of each session, distress ratings were reassessed to monitor symptom change. Each session lasted approximately 40 min, and two sessions were conducted two weeks apart. Written informed consent for treatment and publication was obtained.

In this case, trauma recall and acupoint tapping were intentionally delivered as an integrated, brief intervention. The aim was to assess clinical feasibility and tolerability rather than to isolate the individual contribution of each component.

## 3 Response to treatment

The patient demonstrated rapid and clinically meaningful improvement after two sessions of psychological acupuncture. Therapeutic progress was systematically assessed across multiple domains, including subjective distress, PTSD symptom severity, sleep quality, avoidance behavior, somatic manifestations, and social functioning. The key therapeutic outcomes, including percentage reductions from baseline, are summarized in **Table 1**.

**Table 1:** Timeline and clinical outcomes following psychological acupuncture.

Date/Period	Treatment phase	SUDs	ISI	PCL-5	Percentage change from baseline*	Key clinical notes
Jul 2024	Dog attack	–	–	–	–	Acute panic, nightmares, and avoidance began
Aug 2024–Apr 2025	Pre-treatment baseline	10	23	54	0%	Severe fear, insomnia, hypervigilance
Apr 2025	Session 1	6	16	31	40-43%	Immediate relief; slept ~5 h without nightmares
Jun 2025	Session 2	3	10	25	54-70%	Emotional calmness; resumed short outdoor walks
Jul 2025	1-month follow-up	3	8	22	59-70%	Stable sleep and mood
Sept 2025	3-month follow-up	3	7	20	63-70%	Sustained remission; no adverse events

SUDs, Subjective Units of Distress; ISI, Insomnia Severity Index; PCL-5, PTSD Checklist for DSM-5.

Scale ranges and interpretation.

SUDs 0–10, 0-3 = mild distress, 4-6 = moderate, 7-10 = severe.

ISI 0–28, 0-7 = no clinically significant insomnia, 8-14 = subthreshold, 15-21 = moderate, 22-28 = severe.

PCL-5 0–80, 0-20 = no PTSD, 21-40 = mild-moderate symptoms, 41-60 = moderate-severe, >60 = severe PTSD.

* Percentage reduction ranges reflect changes across SUDs, ISI, and PCL-5 relative to baseline.

After the first session, the Subjective Units of Distress (SUDs) score decreased from 10 to 6. The patient reported immediate physical relaxation and mental clarity and slept approximately five consecutive hours without nightmares that night, compared with her prior fragmented sleep of 2–3 hours.

At the second session two weeks later, baseline SUDs remained at 6 and further decreased to 3 within 15 minutes of intervention. The patient was able to recall the traumatic memory without avoidance or marked distress. Insomnia Severity Index (ISI) scores improved from 23 at baseline to 10, corresponding to a sleep duration of approximately seven hours per night. She also resumed independent outdoor activities for 10–15 minutes without significant anxiety.

Following the two-session intervention, PCL-5 score decreased from 54 to 25. Avoidance behavior and somatic symptoms, including palpitations, tremor, and sweating, were markedly reduced. Improvement was maintained at follow-up, with SUDs scores of 3, ISI scores of 8 and 7, and PCL-5 scores of 22 and 20 at the 1- and 3-month follow-ups, respectively. The patient reported stable sleep, improved emotional regulation, and resumption of daily outdoor and social activities. No adverse events were reported during treatment or follow-up.

Overall, symptom improvement at the 3-month follow-up reflected short-term post-intervention stabilization, while the durability of clinical effects over longer timeframes remains to be determined.

The patient described the intervention as enabling her to confront traumatic memories in a contained manner and to regain a sense of control, particularly with respect to sleep and fear-related symptoms.

## 4 Discussion

### 4.1 Summary of principal findings and relation to current evidence

This case illustrates the clinical application of psychological acupuncture—an integrative mind-body intervention combining guided trauma recall with acupoint tapping—in a patient with severe cynophobia and comorbid PTSD who declined conventional therapy. Following two sessions, improvements were observed across key symptom domains, including subjective distress, avoidance behavior, hyperarousal, and sleep disturbance, with benefits maintained during follow-up.

These observations are consistent with an expanding body of research suggesting that acupuncture and related somatosensory interventions may influence neural circuits involved in fear processing and autonomic regulation. Preclinical studies have demonstrated that stimulation at points such as HT7 (Shenmen) and GV20 (Baihui) can modulate prefrontal-amygdala circuitry and stress-related neurobiological pathways (9, 10). Clinical studies further suggest regulatory effects on hypothalamic-pituitary-adrenal axis activity and autonomic balance, contributing to improvements in emotional stability and sleep (11–13).

Within a broader East-West integrative framework, psychological acupuncture may be viewed as a practice-based approach linking traditional Chinese medicine meridian theory with contemporary psychotherapeutic strategies, offering a culturally consonant option for selected patients unwilling or unable to engage in standard trauma-focused care.

### 4.2 Mechanistic interpretation: bridging neurobiology and TCM theory

Trauma recall in psychological acupuncture differs from conventional trauma-focused psychotherapies in that it is brief, highly structured, and continuously regulated by concurrent somatosensory stimulation. Rather than sustained exposure, recall is limited to core traumatic imagery and paired with acupoint tapping to constrain emotional arousal, thereby reducing the risk of re-traumatization.

The therapeutic effects observed in this case may be understood through complementary neurobiological and traditional Chinese medicine (TCM) frameworks. Psychological acupuncture integrates dual stimulation: cognitive-emotional activation through trauma recall and somatic regulation through acupoint tapping. This combination engages both neural circuits of fear extinction and the energetic networks of Qi regulation, thereby linking psychological processing with bodily homeostasis.

Although psychological acupuncture replaces needle insertion with fingertip tapping, the two modalities share overlapping physiological and theoretical mechanisms. From a neurobiological perspective, both activate cutaneous mechanoreceptors distributed along meridian pathways (11), eliciting afferent somatosensory signals that engage central limbic and regulatory networks (13, 19, 20).

Needle insertion predominantly stimulates Aδ and C fibers, producing deeper somatosensory and neurochemical effects, whereas rhythmic fingertip tapping primarily activates Aβ fibers, generating gentle tactile input that modulates emotional arousal via prefrontal-limbic circuitry (15, 21). Despite differences in stimulus intensity, both methods converge on regulating the HPA axis, enhancing parasympathetic activity, and suppressing amygdala hyperactivation, ultimately facilitating emotional stabilization and fear extinction.

From the perspective of TCM, these two methods are regarded as different manifestations of the same therapeutic principle— “treating the Shen through the meridians (17).” Needle acupuncture focuses on regulating Qi and Blood to harmonize the form, while fingertip tapping emphasizes soothing the spirit and transforming fear. Thus, tapping may be viewed as a non-invasive evolution of acupuncture, retaining its meridian-based regulatory essence while aligning with modern psychotherapeutic principles such as exposure, desensitization, and cognitive reprocessing. The rhythmic tapping on points such as Baihui (GV20), Shenmen (HT7), and Neiguan (PC6) reflects the TCM concept of calming the Shen and harmonizing the Heart and Kidney, which corresponds to modern concepts of emotional regulation and autonomic balance (17, 20).

Taken together, fingertip tapping and needle acupuncture can be seen as operating along a shared mind-body regulatory continuum. Psychological acupuncture represents a contemporary, patient-centered adaptation that extends the scope of acupuncture into the psychological domain, bridging somatic regulation with emotional healing. This approach exemplifies the convergence of ancient and modern paradigms of healing—meridian theory and psychoneuroimmunology—within a unified framework (22, 23).

This multi-layered interpretation suggests that psychological acupuncture exerts bidirectional regulation: top-down modulation of cognitive-emotional processes and bottom-up stabilization through somatic feedback. Neurobiologically, rhythmic tapping may entrain oscillatory activity within prefrontal-limbic networks, facilitating memory reconsolidation and reorganization of fear circuits. From a TCM standpoint, it harmonizes the Heart-Kidney axis, balancing the Shen (spirit) and Jing (essence) to restore internal homeostasis. Together, these mechanisms highlight a dynamic interplay between cognition and physiology, offering a plausible interpretative framework for the symptom changes observed in this case.

### 4.3 Clinical significance and translational value

The most distinctive aspect of this case is the rapidity of therapeutic response. Clinically meaningful changes occurred within two sessions—far shorter than the typical weeks-to-months course required for SSRIs or trauma-focused CBT (9). This suggests psychological acupuncture may serve as a brief, safe, and cost-effective intervention for patients who cannot tolerate standard treatments or require symptom stabilization during acute exacerbations.

The technique’s non-pharmacological, non-invasive, and non-retraumatizing characteristics directly address barriers common in trauma care. Guided recall within a controlled environment, paired with acupoint stimulation, allowed emotional exposure without overwhelming arousal. The absence of adverse effects and high patient acceptability further support its feasibility for outpatient and community use (15).

Beyond individual therapy, psychological acupuncture could be incorporated into integrative psychosomatic clinics or primary care, offering culturally congruent mental health care, particularly in settings where access to psychotherapy is limited. Its low cost, minimal equipment, and short duration make it suggesting potential applicability within integrative clinical settings and interdisciplinary trauma care.

### 4.4 Limitations and directions for future research

As a single case, these results are preliminary. Placebo responses, therapist expectancy, and nonspecific relational factors may have contributed to improvement. Quantitative assessment relied on clinician-administered scales within a single-case design, limiting methodological rigor.

Future research should include randomized controlled trials comparing psychological acupuncture with established modalities such as CBT and EMDR, alongside biomarker studies (e.g., heart rate variability, cortisol, functional neuroimaging) to elucidate its psychoneurophysiological mechanisms (4, 23). Developing standardized acupoint prescriptions and identifying patient subtypes most responsive to this approach will enhance clinical applicability and scientific validity.

Psychological acupuncture should be understood as an emerging, practice-based approach situated at the intersection of traditional Chinese medicine and contemporary trauma-informed care. At this stage, its primary contribution lies in generating clinically grounded observations and informing the design of future systematic studies.

## 5 Conclusions

This case suggests that psychological acupuncture—an integrative mind–body intervention combining guided trauma recall with acupoint stimulation—may represent a feasible and well-tolerated therapeutic option for patients with trauma-related specific phobia and comorbid PTSD who decline conventional pharmacological or trauma-focused psychotherapies.

The rapid symptom stabilization observed in this patient highlights the potential clinical value of pairing controlled cognitive–emotional activation with concurrent somatosensory regulation, particularly in individuals concerned about re-traumatization or adverse effects of standard treatments.

From an integrative medicine perspective, psychological acupuncture offers a structured approach that operationalizes traditional Chinese medicine principles of calming the *Shen* and harmonizing Qi within a contemporary trauma-informed framework.

As a single-case observation, this report does not establish therapeutic efficacy or generalizability. Rather, it provides clinically grounded insight into the feasibility and acceptability of this combined approach and supports the need for future controlled studies to examine its mechanisms, durability of effects, and potential role within stepped or integrative trauma care models.

## 6 Patient perspective

The patient reported experiencing severe fear, sleep disturbance, and social withdrawal following the traumatic dog attack, which significantly affected her daily life and independence. After receiving psychological acupuncture, she noticed a marked reduction in fear intensity and a gradual improvement in sleep quality. She described the treatment as gentle and well-tolerated, allowing her to recall the traumatic experience without feeling overwhelmed. Over time, she regained confidence in going outdoors and reported improved emotional stability without adverse effects.

## Data Availability

The datasets presented in this article are not readily available because of ethical and privacy restrictions. Requests to access the datasets should be directed to the corresponding author.

## References

[1] Wardenaar KJ Lim CCW Al-Hamzawi AO Alonso J Andrade LH Benjet C . The cross-national epidemiology of specific phobia in the World Mental Health Surveys. Psychol Med. (2017) 47:1744–60. doi: 10.1017/S0033291717000174, PMID: 28222820 PMC5674525

[2] Guideline Development Panel for the Treatment of PTSD in Adults, American Psychological Association . Summary of the clinical practice guideline for the treatment of posttraumatic stress disorder (PTSD) in adults. Am Psychol. (2019) 74:596–607. doi: 10.1037/amp0000473, PMID: 31305099

[3] Schrader C Ross A . A review of PTSD and current treatment strategies. Mo Med. (2021) 118:546–51. PMC8672952 34924624

[4] Jia Y Ye Z Yang F Chai J Xu H Yang J . Pharmacotherapy for post-traumatic stress disorder: systematic review and meta-analysis. Ther Adv Psychopharmacol. (2025) 15:20451253251342628. doi: 10.1177/20451253251342628, PMID: 40529797 PMC12171264

[5] Hudays A Gallagher R Hazazi A Arishi A Bahari G . Eye movement desensitization and reprocessing versus cognitive behavior therapy for treating post-traumatic stress disorder: A systematic review and meta-analysis. Int J Environ Res Public Health. (2022) 19:16836. doi: 10.3390/ijerph192416836, PMID: 36554717 PMC9778888

[6] Bisson JI Berliner L Cloitre M Forbes D Jensen TK Lewis C . The international society for traumatic stress studies new guidelines for the prevention and treatment of posttraumatic stress disorder: methodology and development process. J Trauma Stress. (2019) 32:475–83. doi: 10.1002/jts.22421, PMID: 31283056

[7] Wright S Karyotaki E Cuijpers P Bisson J Papola D Witteveen AB . Predictors of study dropout in cognitive-behavioural therapy with a trauma focus for post-traumatic stress disorder in adults: An individual participant data meta-analysis. BMJ Ment Health. (2024) 27:e301159. doi: 10.1136/bmjment-2024-301159, PMID: 39537555 PMC11580285

[8] Song K Xiong F Ding N Huang A Zhang H . Complementary and alternative therapies for post-traumatic stress disorder: A protocol for systematic review and network meta-analysis. Med (Baltimore). (2020) 99:e21142. doi: 10.1097/MD.0000000000021142, PMID: 32664144 PMC7360199

[9] Stapleton P Kip K Church D Toussaint L Footman J Ballantyne P . Emotional freedom techniques for treating post traumatic stress disorder: an updated systematic review and meta-analysis. Front Psychol. (2023) 14:1195286. doi: 10.3389/fpsyg.2023.1195286, PMID: 37637920 PMC10447981

[10] Kwak HY Leem J Seung HB Kwon CY Jeong HS Kim SH . Acupuncture therapy for military veterans suffering from posttraumatic stress disorder and related symptoms: A scoping review of clinical studies. Healthcare (Basel). (2023) 11:2957. doi: 10.3390/healthcare11222957, PMID: 37998449 PMC10671227

[11] Tang X Lin S Fang D Lin B Yao L Wang L . Efficacy and underlying mechanisms of acupuncture therapy for PTSD: evidence from animal and clinical studies. Front Behav Neurosci. (2023) 17:1163718. doi: 10.3389/fnbeh.2023.1163718, PMID: 37200784 PMC10187757

[12] Zhang J Shen QH Lin X Liu T Li Y Yu Y . Transauricular vagus nerve stimulation in preventing post-traumatic stress disorder in emergency trauma surgery patients in China: a study protocol for a multicenter, double-blind, randomised, controlled trial. BMJ Open. (2025) 15:e093467. doi: 10.1136/bmjopen-2024-093467, PMID: 39832985 PMC11749037

[13] Bae R Kim HK Lu B Ma J Xing J Kim HY . Role of hypothalamus in acupuncture’s effects. Brain Sci. (2025) 15:72. doi: 10.3390/brainsci15010072, PMID: 39851439 PMC11763592

[14] Wang Q Wang D Lv Y Li Q . Traditional chinese medicine in the management of anxiety disorders: A narrative review of theoretical foundations, clinical applications, and modern integrative approaches. Neuropsychiatr Dis Treat. (2025) 21:1215–33. doi: 10.2147/NDT.S535646, PMID: 40548351 PMC12182740

[15] Wittfoth D Beise J Manuel J Bohne M Wittfoth M . Bifocal emotion regulation through acupoint tapping in fear of flying. NeuroImage Clin. (2022) 34:102996. doi: 10.1016/j.nicl.2022.102996, PMID: 35378497 PMC8980501

[16] National Health Commission of the People’s Republic of China . Technical Specifications for Traditional Chinese Medicine Psychological Therapies (2023 Edition). Beijing: National Health Commission (2023).

[17] Yang YD Zhong W Chen M Tang QC Li Y Yao LL . Electroacupuncture alleviates behaviors associated with posttraumatic stress disorder by modulating lipocalin-2-mediated neuroinflammation and neuronal activity in the prefrontal cortex. J Integr Med. (2025) 23:537–47. doi: 10.1016/j.joim.2025.07.002, PMID: 40695649

[18] WHO Regional Office for the Western Pacific . WHO Standard Acupuncture Point Locations in the Western Pacific Region. Manila: World Health Organization (2008).

[19] Grant S Colaiaco B Motala A Shanman R Sorbero M Hempel S . Acupuncture for the treatment of adults with posttraumatic stress disorder: A systematic review and meta-analysis. J Trauma Dissociation. (2018) 19:39–58. doi: 10.1080/15299732.2017.1289493, PMID: 28151093

[20] Freedom J Warner J Hux M . Research on acupoint tapping therapies proliferating around the world. Energy Psychol. (2022) 14:22–37. doi: 10.9769/EPJ.2022.14.1.JF, PMID: 12230991

[21] Feinstein D . Integrating the manual stimulation of acupuncture points into psychotherapy: A systematic review with clinical recommendations. J Psychother Integr. (2023) 33:47–67. doi: 10.1037/int0000283, PMID: 41574093

[22] Chen CY Zhang Y . Acupressure as a non-pharmacological treatment for depression: Neurophysiological, biochemical, and psychological mechanisms. J Psychiatr Res. (2025) 190:32–46. doi: 10.1016/j.jpsychires.2025.07.036, PMID: 40753796

[23] Assouline A Mendelsohn A Reshef A . Memory-directed acupuncture as a neuromodulatory treatment for PTSD: Theory, clinical model and case studies. Transl Psychiatry. (2022) 12:110. doi: 10.1038/s41398-022-01876-3, PMID: 35296636 PMC8927413

